# Skewed performance distributions as evidence of motor constraint in sports and animal displays

**DOI:** 10.1098/rsos.230692

**Published:** 2023-11-08

**Authors:** David M. Logue, Tyler R. Bonnell

**Affiliations:** ^1^ Department of Psychology, University of Lethbridge, Lethbridge, Alberta, Canada; ^2^ Department of Biology, University of Puerto Rico Mayaguez, Mayaguez, Puerto Rico

**Keywords:** bird song, display signals, motor performance, sexual selection, skew, sports performance

## Abstract

Animal displays (i.e. movement-based signals) often involve extreme behaviours that seem to push signallers to the limits of their abilities. If motor constraints limit display performance, signal evolution will be constrained, and displays can function as honest signals of quality. Existing approaches for measuring constraint, however, require multiple kinds of behavioural data. A method that requires only one kind could open up new research directions. We propose a conceptual model of performance under constraint, which predicts that the distribution of constrained performance will skew away from the constraint. We tested this prediction with sports data, because we know *a priori* that athletic performance is constrained and that athletes attempt to maximize performance. Performance consistently skewed in the predicted direction in a variety of sports. We then used statistical models based on the skew normal distribution to estimate the constraints on athletes and displaying animals while controlling for potential confounds and clustered data. We concluded that motor constraints tend to generate skewed behaviour and that skew normal models are useful tools to estimate constraints from a single axis of behavioural data. This study expands the toolkit for identifying, characterizing, and comparing performance constraints for applications in animal behaviour, physiology and sports.

## 1. Introduction

Physiology and physics conspire to prevent animals from running as fast as a speeding bullet, exerting as much power as a locomotive, or leaping over a tall building. In the language of behavioural ecology, constraints (e.g. on force production, speed) limit motor performance. Constraints are particularly important in the evolution of animal communication, where they can stabilize display-based signalling systems like songs and dances [1,2]. According to honest signalling theory, communication systems in which signal senders and signal receivers have different fitness interests tend to break down over evolutionary time unless costs or constraints enforce reliable signalling. There is evidence that constraints stabilize signalling systems in spiders [3,4], frogs [5], birds [6], mice [7], bats [8], and primates [9–11]. Although most studies have focused on male signallers and acoustic displays, there is also evidence of constraints in female signallers [11,12] and visual displays [10,13,14].

Constraint and motor performance are closely linked concepts in the animal communication literature. Some authors define performance as an adaptive approach to a behavioural limit [15,16]. Another common definition of performance centres on the degree of challenge the animal experiences when performing the behaviour [17]. It would be useful to unify these two definitions, and to formalize the heretofore vague term ‘challenge’. Performance constraints can limit performance in any taxon that performs displays, but much of the research on display performance has come from the bird song literature. We attribute this bias to the ease of measuring the acoustic properties of song, and the pioneering role of ornithologist Jeffrey Podos, whose approach to identifying constraints [18] has had a major influence on the study of constrained display performance.

Most New World sparrow (family: Emberizidae) songs include trills, which require the singer to repeat a short vocal unit in rapid succession (fig. 1 in [18]). Podos measured the rate of syllable production (trill rate) and the difference between the maximum and minimum fundamental frequency (frequency bandwidth) in a set of sparrow trills. Plotting the data on a scatterplot reveals a triangular distribution (fig. 5 in [18]). The upper right quadrant of the scatterplot, which represents songs with high trill rates and high frequency bandwidths, is devoid of data. Podos concluded that trill rate and frequency bandwidth ‘trade-off’ against one another at the limit of performance owing to a motor constraint. He treated the edge of the distribution that abuts the vacant quadrant as an estimate of the song structures that require birds to most closely approach the limits of motor performance and proposed that the orthogonal distance between this ‘performance limit’ and a plotted trill could serve as an inverse metric of performance called ‘vocal deviation’ [19]. In the intervening decades, numerous studies followed this approach to identify performance limits and measure performance in bird songs and other acoustic displays [6,20]. However, some scholars question whether motor constraints are the cause of the observed trade-offs.

Kroodsma [21], in particular, criticized Podos's methods. He argued that because songbirds learn their songs from adult birds, the observed limits of song structure are a consequence of cultural (i.e. learned) constraints on song development rather than motor constraints on song production. This critique raises an important question that applies to all kinds of constrained behaviour in animals: what kind of evidence can reveal that a motor constraint limits performance? All behaviour is constrained, and all distributions of behaviour are limited, but it is not necessarily true that the observed limit of a behaviour is enforced by a motor constraint [18,22,23]. Alternative hypotheses are that selection has not favoured phenotypes that approach the performance limit or that there has not yet been sufficient time for selection to drive performance to its limit [23].

The question of whether a display approaches a performance limit is important for several reasons. First, if displaying individuals do not approach a performance limit, a constraint-based mechanism cannot explain honest signalling (such systems may be stabilized by other mechanisms; [1,24,25]). Second, if animals do not approach a limit of performance, it is not possible to use observed displays to characterize performance limits, or to measure performance as the deviation from those limits. Third, displays that consistently approach a performance limit suggest the existence of selection to perform at a high level. In the case of male sexual displays, that selection comes from females’ preferences for high performance. Theory suggests females evolve to prefer displays that reliably indicate male quality [1]. If displays do not consistently approach a performance limit, it is reasonable to conclude that selection for high performance is not the dominant evolutionary force shaping the display. In such cases, it is possible that the optimum display level is lower than the maximum possible, such that a higher performance display would be wasteful, or even perceived as suboptimal or abnormal by receivers (i.e. the trait is under stabilizing selection).

In this paper, we attempt to evaluate and expand the toolkit for identifying, characterizing, and comparing performance constraints. Our approach emerges from behavioural ecology, but we hope that the methods we develop will find purchase in other disciplines. We begin with a review of existing methods to identify constraints in animal displays. We then propose a conceptual model of performance under constraint that generates the prediction that the distribution of performance under constraint will skew away from a nearby constraint. We test this prediction with data from sports and bird song. Finally, we demonstrate a powerful skew-based approach to empirical modelling that uses a single dimension of behavioural performance to test for and characterize constraints while accounting for confounds and clustered data.

## 2. Evidence that displays approach performance limits

Our literature review revealed seven kinds of evidence that animal displays approach performance limits (for alternative classifications, see [17,25]). We attempted to review the full breadth of animal display performance research. Our examples, however, are strongly biased towards bird song, owing to a bias in the display performance literature. Findings from bird song studies may not apply to other taxa and signalling modalities. Here, however, we are interested in the approaches used to detect and estimate constraints, which can be generalized across systems.

### 2.1. Extreme performance

The simplest evidence of performance constraint is *a priori* knowledge that a behaviour is extreme relative to similar behaviours. Extreme display parameters in the literature include acoustic amplitude [11,26–28], density (i.e. the proportion of an acoustic signal in which the animal is vocalizing) [26,29], frequency [30,31] and frequency modulation (FM; [16,32,33]), coordination among moving body parts [34], movement amplitude [10], power [13,35], speed [10,31] and vigor [4,13]. Studies of display performance typically combine *a priori* expectations of constrained performance with other kinds of evidence.

Constraints operate on the mechanisms of behaviour, but they have measurable effects on the behaviour itself [17]. Ideally, studies of extreme display performance would link constrained physiological processes to constraints at the level of the display by monitoring physiological performance in displaying animals. Only a few studies, however, take such an integrative approach (e.g. [14,34]). More common are studies that infer mechanistic constraint from extreme performance. A strong version of this approach identifies behaviours that are so extreme they are likely to approach a performance limit. For example, the songs of the white bellbird (*Procnias albus*) are the loudest bird vocalizations known to science [28]. A less compelling, but still useful, approach relies on behaviour for which the locus of the constraint is unknown, but the direction of constraint seems clear. Examples of this kind of inference include studies of display consistency [36–40]. Consistency, or the ability to limit the variance in display parameters, can be constrained even if the parameters of the display are not constrained.

### 2.2. Trade-offs attributable to allocation constraints

Podos's use of trade-offs to identify performance constraints was an important innovation in the study of display performance [18]. This method attempts to identify trade-offs generated by allocation constraints, which occur when two measured attributes both rely on some common resource [23]. At the limit of performance, the organism maximizes its use of the shared resource, such that an increase in one attribute necessitates a decrease in the other. Many studies have tested for the trade-off that governs the relationship between frequency bandwidth and trill rate in bird songs (e.g. [8,12,41–46]). Others studies test for different kinds of allocation trade-offs in bird songs [16,28,33,47,48] and other animal displays [5,7–9].

Once performance limits have been estimated, it is possible to estimate the performance of a behavioural event in terms of the deviation (orthogonal distance) of that event from the limit line. An important limitation of the trade-off approach is that it requires two measurable display attributes that rely on a common resource. Another limitation concerns the statistical methods used to estimate the performance limit. Several approaches have been proposed [18,49,50], but none of these are robust to variation in the shape of the data cloud [16,51].

### 2.3. Development of performance

Motor performance typically becomes less constrained as an individual matures from infancy to adulthood. Later, motor performance becomes more constrained as the individual senesces from adulthood to old age. If display behaviours require animals to approach performance constraints, display performance should increase as an animal matures and decrease as it senesces. Several studies of vocal signals support one or both predictions [11,37,38,44,46,52–54]. Motor performance can also improve with practice over shorter time scales if ‘warming up’ shifts the performance limit upwards [51,55–57]. Finally, if animals learn constrained behaviours, it is possible to tutor them with supernormal stimuli. When Podos did this with bird songs, the subjects modified the songs in ways that lowered their motor performance requirements, suggesting that the demands of the stimuli exceeded their motor capacity [58].

### 2.4. Evolutionary

If a trait that improves performance in a non-signalling domain also reduces signalling performance, there is a functional conflict that can generate a correlation between the trait and performance [23]. Among-species correlations between display performance and either overall body size or the size of specific body parts can be evidence that size constrains performance [3,19,45,47,59]. Higher-than-expected performance from a species in a taxon with a functional conflict may suggest that the focal species' lineage has evolved one or more adaptations to overcome the ancestral constraint [60,61]. Similarly, extreme ‘morphological, mechanical or physiological adaptations’ that underlie displays are evidence of constraint because they indicate that selection for extreme performance has driven the evolution of performance limits [25, p. 211].

### 2.5. Current directional selection for high performance

Open-ended directional selection on a heritable display trait will eventually drive performance to its limit, at which point performance will be constrained [25]. Numerous studies have tested whether putatively constrained display traits are subject to selection from potential mates [7,10,14,15,30] and competitors [31,62–65].

### 2.6. Correlations with sender attributes

Correlations between individuals' physical traits (e.g. body size, body condition, age) and display performance can be evidence that the physical trait, or trait(s) that are correlated with it, constrains performance (e.g. [3,8,52,66]).

### 2.7. Experimental manipulations of performance

Manipulating animals in ways that are expected to depress or elevate performance can provide insights into performance constraints [22]. We are aware of only one study that applies this approach to animal displays [67].

## 3. Skewed performance as evidence of constraint

Our survey of methods to identify and characterize performance constraints reveals that none of the established methods can characterize constraints from a single behavioural trait measured from a single population. A method that only requires data from one behavioural trait could expand the study of performance constraints into traits that are not subject to known allocation trade-offs.

A previous study of a large bird song dataset showed that the distributions of three deviation scores skewed away from their respective performance constraints [16]. The authors interpreted this pattern as evidence of constraint:

… if a performance limit constrains the structure of vocalisations and singers are attempting to sing with high performance (at least under some circumstances), vocalisations should cluster near the limit and deviation scores should skew away from the limit. This prediction views performance limits as inducing a ‘ceiling effect’ that truncates one tail of the distribution [16, p. 712].

The next section elaborates on this hypothesis.

## 4. A conceptual model of performance under constraint

**Figure 1.**
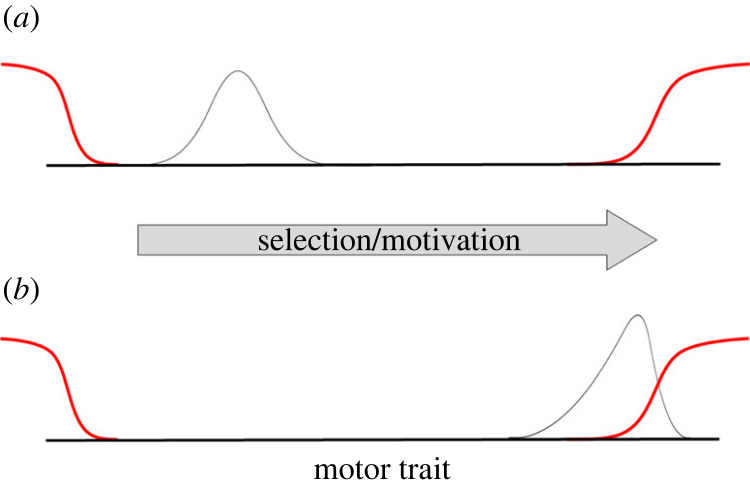
A model of the evolution of performance under constraint. The *x*-axis represents a motor trait, the grey curves are distributions of that motor trait, and the red curves describe the degree of constraint on the motor trait. (*a*) An unconstrained trait does not interact with either constraint curve. Selection (at the population level) or motivation (at the individual level) could shift the distribution of the motor trait until (*b*) it encounters a constraint. The constraint acts like a penalty on performance, skewing the distribution. Figure is not to scale (see the electronic supplementary material, figure S1 for a calculated representation of the skew normal distribution).

Our conceptual model is set in motor trait space (figure 1). The motor trait space is bound by constraints on one or both sides [68]. Following Podos [25], the constraints are modelled as ramps, to indicate that the constraint intensifies as the motor trait becomes more extreme. When a population is far from a constraint, the constraint has no effect on motor performance or signal structure [17] and performance is distributed normally (figure 1*a*). Although behaviour is not constrained in this scenario, it may incur costs [25]. Directional selection on display performance can drive populations to evolve into constrained areas of the motor trait space [25,69]. When that happens, the model treats constraints as penalties on performance.

The key prediction is that the distribution of the trait when it is under constraint (i.e. performance) will be skewed, such that the long tail of the distribution points away from the constraint (figure 1*b*). All else equal, the degree of skew should correspond to the degree of constraint. If we assume that motor performance covaries with display performance in an approximately linear manner, the model predicts that behavioural traits under constraint will tend to skew away from the constraint.

The model can also be applied at the individual level. In this version, unconstrained individuals exhibit normal distributions of the behaviour (figure 1*a*). When individuals attempt to increase their performance, they encounter their own personal constraint, which skews the distribution of their performance (figure 1*b*). Based on this model, we predict that both population-level and individual-level performance distributions will tend to skew away from constraints.

## 5. Methods: testing for skew in athletes' and songbirds’ performance

The ideal dataset to test the prediction that distributions of constrained performance tend to skew away from the constraint would have the following properties: (i) performers are both constrained and motivated to perform high, such that the distribution interacts with the constraint (figure 1); (ii) performers belong to a single statistical population. Mixing populations (e.g. males and females, juveniles and adults) probably violates the assumption that the distribution would be normal in the absence of constraints; (iii) the direction of the relevant constraint should be clear *a priori*; (iv) large datasets are desirable because they are robust to sampling error; and (v) the measured behavioural traits should be closely related to the underlying constrained mechanism. Other variables that influence the behaviour will add statistical noise [16,17,20,70], which will tend to weaken the skew of the performance distribution. We will relax conditions (ii) and (v) later in the paper when we introduce a modelling technique that allows statistical controls for clustering and covariates.

We tested the prediction that constrained performance skews away from the constraint with data from high-level sports. Athletic performance is a natural comparator to animal displays because performers in both domains approach the limits of performance, improve with practice [40], warm up [51,55–57], and get ‘rusty’ from lack of practise [40]. We chose sports datasets that met the five criteria listed above: (i) we know high-level athletes are trying to perform high because they are motivated by competition, glory and financial reward. There is abundant *a priori* evidence that athletic performance is constrained [71]; (ii) we chose datasets that separate data from men and women. Most of these datasets are restricted to elite athletes (Olympics), but one includes a wider range of advanced athletes (marathon); (iii) in athletic performance, the direction of the relevant constraint is obvious: race time is constrained on the low end, whereas jump distance and throwing distance are constrained on the high end; (iv) sports generates large, iterated datasets; and (v) we analysed types of athletic performance that were closely related to the underlying constrained mechanisms. In addition to the sports data, we analysed a bird song dataset from a recent study of vocal performance [16].

We downloaded the results of the Boston Marathon from 2015–2019 from GitHub [72]. Cases comprise all marathon finishers. We used the variables *finish time* and *gender*. Data from the Olympic Games were downloaded from Kaggle.com [73]. The full dataset comprises Summer and Winter Olympic results from 1896–2022. We generated datasets for each of the following events: 100 m dash, 10 000 m run, long jump, shotput and 100 m freestyle swim. The number of non-medalists in the dataset varied across years, so we filtered out non-medalists to leave three data points for each event from each Olympics. We used the variables *finish time* (100 m dash, 10 000 m run, 100 m freestyle swim) and *distance* (long jump, shotput). Olympic data were separated by gender but pooled across years because there were only three values from each year.

We analysed three composite acoustic features (deviation scores) derived from male Adelaide's warblers (*Setophaga adelaidae*) songs. Trade-off analyses suggest these features measure constrained vocal performance [16,51]. Subjects were nine free-living, individually colour-banded, mated males. In 2012, observers continuously recorded these males during the first 3.5 h of activity for four days each. Adelaide's warblers sing repertoires of discrete song types, each of which is a frequency modulated trill. Each song comprised 23.5 ± 1.8 notes (represented by continuous traces on a spectrogram), separated by brief silent gaps. Song types were manually classified from sound spectrograms for each individual, which is a straightforward and highly repeatable process in this species [51]. Song recordings that produced clear spectrograms with minimal overlapping noise were subjected to acoustic analysis with Luscinia v2.14 [51,74].

Luscinia was used to extract the following acoustic features from each note: start time, end time, maximum peak frequency, minimum peak frequency, beginning peak frequency and end peak frequency. We used these features to calculate four simple acoustic variables: note duration (end time–start time), gap duration (start time of a note–end time of the prior note), note frequency bandwidth (maximum peak frequency/minimum peak frequency), and gap frequency bandwidth (the greater of the previous note's end peak frequency and the subsequent note's beginning peak frequency/the lesser of those two values) [51,75]. We then used mixed quantile regression (tau = 0.05) to estimate performance limits with the trade-off method while accounting for variation attributable to individuals. We found statistically significant slopes for the limits of note duration versus gap duration, note duration versus note bandwidth, and gap duration versus gap bandwidth. We calculated deviation scores as the orthogonal distance of each note from the regression line. We refer to these deviation scores as recovery time (note duration versus gap duration; estimates the ability to sing songs with long notes and short silent gaps), voiced FM (note duration versus note bandwidth; estimates the speed of FM during note production), and unvoiced FM (gap duration versus gap bandwidth; estimates the speed of ‘FM’ between notes). For all three deviation scores, lower values indicate higher performance. For further details on the birdsong data, see [51].

We plotted the distributions as histograms and measured their skewness. Skewness was measured using the Fisher's moment coefficient of skewness (µ_3_/*σ*^3^) from the e1071 package in R [76]. Bootstrapped samples were taken to provide 95% confidence intervals (CIs) for each skewness measure (electronic supplementary material, table S1). Based on our conceptual model, we predicted that the distributions of finish times for the marathon, 100 m dash, 10 000 m run and 100 m freestyle would be positively skewed because the ability to finish fast (short time) is constrained. We predicted that the bird song metrics would also skew positively because the ability to sing with high vocal performance (i.e. achieve a low deviation score) is constrained. We predicted the distribution of the other sporting results would be negatively skewed because athletes' capacities to jump far and throw far are constrained.

## 6. Results: testing for skew in athletes' and songbirds’ performance

**Figure 2.**
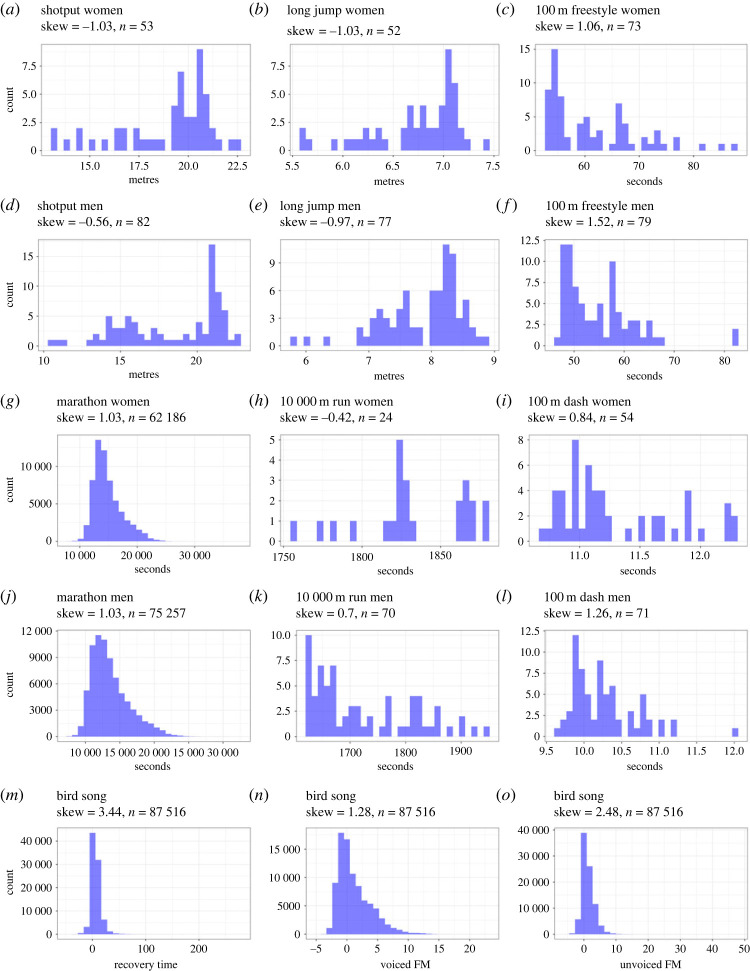
Sports histograms and skewness for raw data from: (*a*) women's shotput, (*b*) women's long jump, (*c*) women's 100 m freestyle swim, (*d*) men's shotput, (*e*) men's long jump, (*f*) men's 100 m freestyle swim, (*g*) women's marathon, (*h*) women's 10,000 m run, (*i*) women's 100 m dash, (*j*) men's marathon, (*k*) men's 10 000 m run, (*l*) men's 100 m dash, (*m*) bird song recovery time, (*n*) bird song voiced FM, (*o*) bird song unvoiced FM.

Performance data consistently skewed away from the direction of constraint in all tested sports activities and across genders except for the women's 10 000 m run, which was not in the predicted direction (figure 2). Notably, the women's 10 000 m run had our lowest sample size (*n* = 24), the data included influential outliers, and the magnitude of the skewness (−0.42) was low compared the other examples (|skewness| < 0.5). The three bird song performance measures skewed in the expected direction.

## 7. Methods: modelling performance with the skew normal distribution

### 7.1. Skew normal distribution

Simple measures of skewness, like we used above, will not measure constraint accurately if underlying data structure or confounding variables influence the skewness of a distribution (see Discussion). Here, we propose a modelling approach to estimate the shape of constraints that drive skewness while controlling for data structure and confounding variables. Based on our assumptions that (i) the distribution of performance is normally distributed in the absence of constraint, and (ii) constraint causes the distribution of performances to skew away from the constraint, the skew normal distribution is an ideal candidate to build a model.

The skew normal distribution is based on an interaction between a normal distribution and a cumulative distribution function (CDF). It takes three parameters: the mean of the population (*µ*), the standard deviation of the population (*σ*) and alpha (*α*) which can be interpreted as the magnitude of the skew:

7.1 $$\mathrm{skew}\;\mathrm{normal}\;(y|\mu ,\sigma ,\alpha )=\overset{\mathrm{normal}\;\mathrm{distribution}}{\overbrace{\frac{1}{\alpha \sqrt{2\pi }}\exp\left(-\frac{1}{2}{\left(\frac{y-\mu }{\sigma }\right)}^{2}\right)}}\times \overset{\mathrm{cumulative}\;\mathrm{distribution}\;\mathrm{function}}{\overbrace{\left(1+\mathrm{erf}\left(\alpha \left(\frac{y-\mu }{\sigma \sqrt{2}}\right)\right)\right)}}.$$

From the equation above, the skew normal distribution can be seen to be a normal distribution (with mean *µ*, standard deviation *σ*) multiplied by a CDF that is calculated using the error function (erf) with a scaling parameter *α*. This error function takes a sigmoidal shape. For the purpose of estimating a constraint line we invert and normalize the values from the CDF (*V*_CDF_). By inverting and normalizing the CDF, constraint values (*V*_constraint_) range between 0 and 1, where higher values indicate more constrained behaviour:

7.2 $$V_{\mathrm{constraint}}=\frac{\left(\max(V_{\mathrm{CDF}})-V_{\mathrm{CDF}}\right)}{\left(\max(V_{\mathrm{CDF}})-\min(V_{\mathrm{CDF}})\right)}.$$

The combination of the normal distribution and the constraint curve can then be used to describe the shape of a wide range of skewed distributions (electronic supplementary material, figure S1).

Assessing constraint with a statistical model, rather than simply measuring skewness, allows us to account for variables that might influence the constraint line. For example, it is possible to build a model that measures how the constraint curve has shifted over years (e.g. alpha∼year), and uses random terms to account for repeated measures within individuals (e.g. alpha∼year + (1|ID)). As the constraint line is a function of not only alpha but also of mu and sigma, we also show how once the model is fit it can be used to quantify and visualize how the constraint line shifts based on covariates (e.g. year).

### 7.2. Assumptions

The assumptions of these kinds of models are straightforward. Samples are assumed to be independent, but random terms can be used when data are clustered and time series approaches can be used when samples are autocorrelated. Distributions of behaviour are assumed to be normal in the absence of constraint, and residuals are assumed to be homoscedastic. Perhaps the most important assumption is that the constraint is sigmoidal. It is not yet clear whether a sigmoidal shape best characterizes most constraints. Users should attend to the model fit with validation tools like posterior predictive checks.

### 7.3. Simulation example

**Figure 3.**
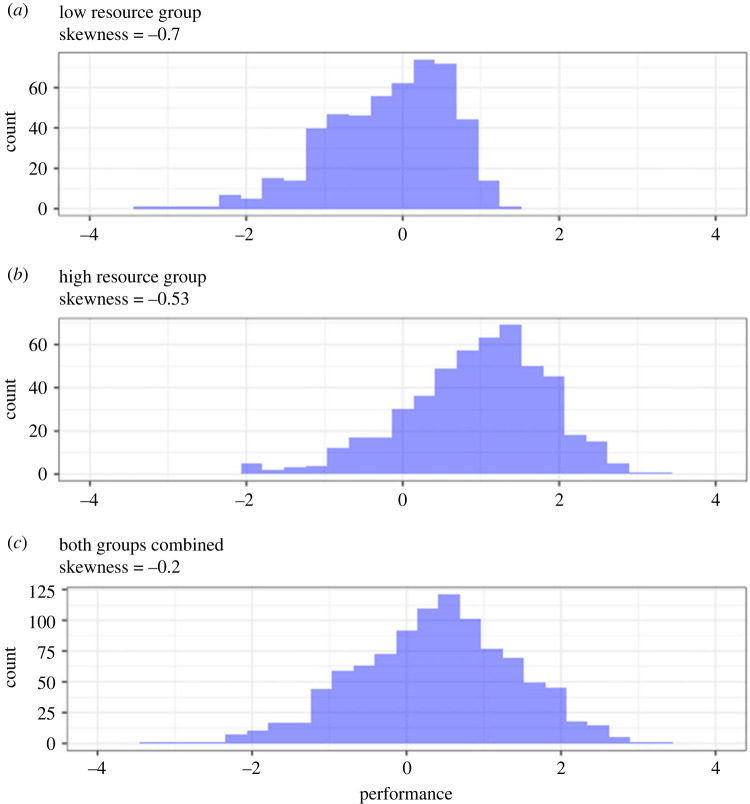
Simulated data of performance under constraint. Data represent behavioural observations from (*a*) a low resource environment, (*b*) a high resource environment and (*c*) both environments combined.

We used simulated data to demonstrate how models based on the skew normal distribution estimate constraints (figure 3). To generate these data, we assumed an individual performs a behaviour with some target level of performance that can be measured. We used *b* as a measure of this performance and generate performance measures for this individual by drawing values from a normal distribution with mean = 0 and standard deviation = 1. We then constrained performance above the target mean by applying a penalty that grows as the performance increases above the mean: *b*/(1 + 0.5 × (*b-*mean)). Finally, we introduced a confound by varying the target performance according to resource availability. Compared to the low resource condition, the high resource condition has higher target performance (i.e. mean = 1) and a weaker penalty: *b*/(1 + 0.1 × (*b*-mean)). Data and relevant code for this research work are stored in GitHub (github.com/tbonne/skewnormal) and have been archived within the Zenodo repository: https://zenodo.org/record/8349782.

**Figure 4.**
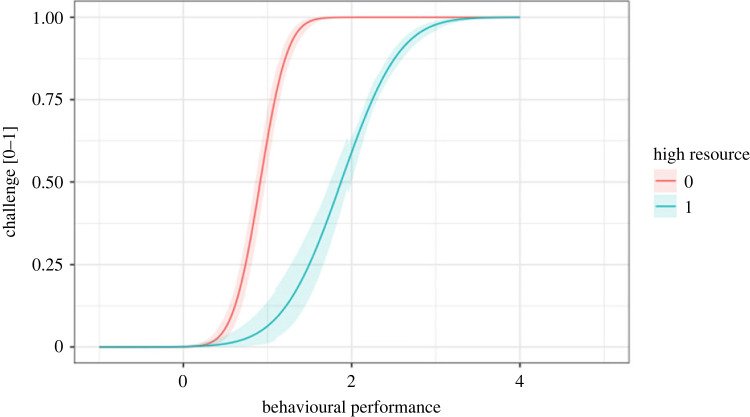
Estimated constraint lines for behavioural performance under high and low resource conditions. Lines are the output of an analysis of simulated data using a skew normal model.

**Figure 5.**
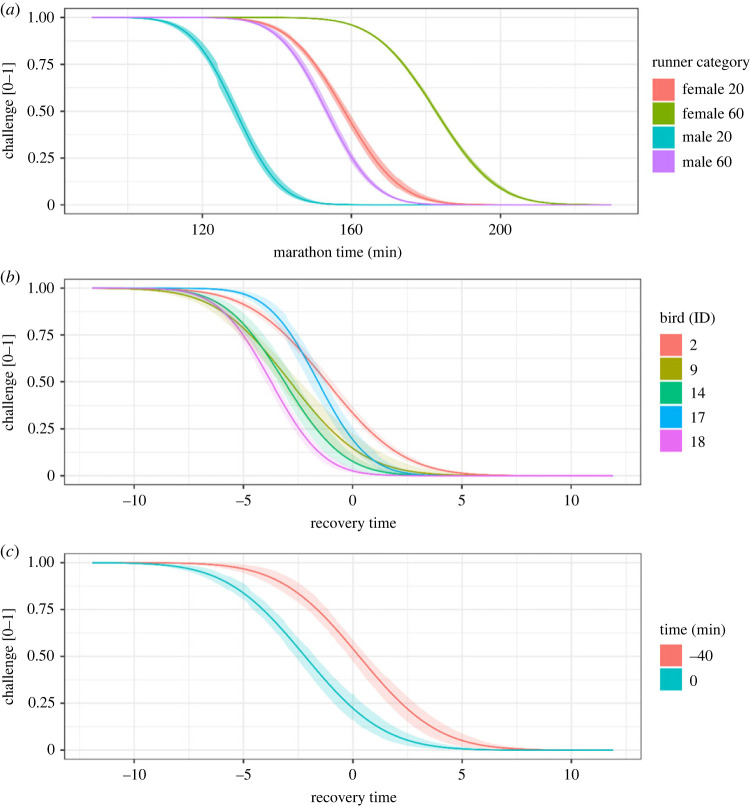
Constraint curves extracted from skew normal distribution models: (*a*) plot of the constraint curve for a 20-year-old male/female, and 60-year-old male/female, (*b*) constraint curves for five birds, and (*c*) constraint curve for bird 2 at different times of the day. The five birds selected for (*b*) were chosen because they had the largest samples (greater than 6000 notes per bird). Shaded areas represent 95% credible intervals.

**Figure 6.**
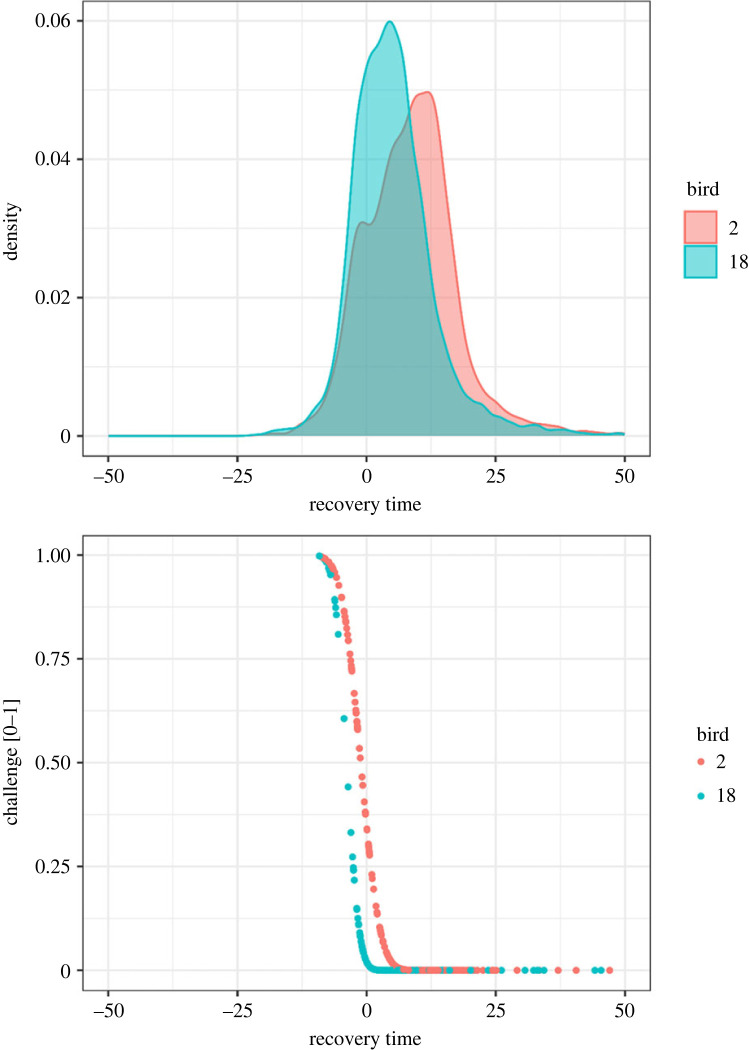
Vocal performance in two individual birds: (*a*) a density plot of the performance metric recovery time (lower values indicate higher performance), and (*b*) individual songs plotted on the two birds' constraint curves.

Skewness was moderate to high in the low and high resource groups, but combining the groups produced a distribution with low skew (skewness = −0.20; 95% CI: −0.33, 0.04). If one were to observe this combined distribution in nature and simply measure its skewness, they might incorrectly conclude that the behaviour is not constrained. We used a skew normal distribution to estimate the constraint on behavioural performance while accounting for the confounding effect of resource level (alpha∼resource condition). We then estimated alpha as a function of the resource condition (table 1), and plotted constraint lines to visualize the effect of resources on performance (figure 4). These constraint lines can be used to estimate the degree of challenge that the actor experiences when it performs a behaviour. The challenge is described by the *y*-axis on a constraint curve, such that more extreme performance incurs greater challenge (figures 5 and 6).

**Table 1.** Parameter estimates from a model based on the skew normal distribution.

	estimate	l-95% CI	u-95% CI
intercept	−0.16	−0.23	−0.10
sigma_intercept	−0.17	−0.22	−0.12
alpha_intercept	−5.45	−6.86	−4.20
resource_high	1.11	1.02	1.21
alpha_high	2.58	1.26	3.90

*Intercept* estimates the mean behavioural performance, *sigma_intercept* estimates the standard deviation, and *alpha_intercept* estimates the magnitude of skew. The parameter *resource_high* estimates the change in mean performance under high resource conditions, and *alpha_ high* estimates how the skew parameter changes under high resource conditions.

The analysis successfully recovered the key attributes of the simulated data. It accurately showed that the constraint line is steeper and starts at a lower level of behavioural performance under the low resource condition (figure 4). We conclude that our modelling approach works as intended. Importantly, it allowed us to control for statistical confounds, revealing evidence of constraint that would have been missed with simple measures of skewness.

We ran a second series of simulations to better understand how sample size affects estimations of alpha (see the electronic supplementary material for details). Based on these simulations, we concluded: (i) Hamiltonian Monte Carlo can be used to estimate alpha even at low sample sizes, but (ii) estimates are biased towards zero when sample sizes are low (i.e. priors centered on zero play a larger role); (iii) using credible intervals improves inference; and (iv) larger sample sizes are needed to detect smaller differences in skewness.

### 7.4. Analysis

We made models with the dependent variables ‘finish time’ from the 2014 Boston marathon dataset and the performance metric ‘recovery time’ from the bird song dataset to demonstrate the ability of the skew normal distribution to model constraint. We used the marathon data to model the effects of age and gender on the constraint: alpha∼age + gender. For the bird song model, we used data before dawn and accounted for time relative to sunrise, as well as the song identity (ID) in which the note-level recovery time was measured. Finally, we used a random intercept for individuals to estimate the constraint faced by each bird: alpha∼time + (1|birdID/songID). By including both bird ID and song ID we also account for repeated measures within bird and within songs. To extract the constraint line from the marathon and bird song model, we used the posterior estimates of µ, *σ* and *α* to calculate the cumulative distribution function (equation (7.1)) and converted it to a constraint line using equation (7.2). All data and R code are available at (github.com/tbonne/skewnormal).

Based on the hypothesis that our constraint curves measure motor constraints, we predicted that younger runners and men would have more extreme (less restrictive) constraints than older runners and women. Based on previous findings that Adelaide's warblers’ vocal performance improves (i.e. they warm up) over the course of morning singing [51,55], we predicted that the constraints on birds' recovery time performance would be less extreme (more restrictive) when they first begin singing, compared to after the dawn chorus.

## 8. Results: modelling performance with the skew normal distribution

### 8.1. Posterior predictions of the constraint line

Leave one out cross validation (loo-cv) showed that a model of the 2014 Boston marathon finishing times that accounts for age and gender effects outperforms a simple model without age and gender [77]. The simple model was estimated to have lower out-of-sample accuracy, measured by the expected log pointwise predictive density (elpd_diff = −35.2, se_diff = 9.1). The same was true when using a normal distribution instead of a skew normal (elpd_diff = −6219.6, se_diff = 90.6). Posterior predictions showed that relative to the constraint curves of older (60 years old) runners and women, younger runners’ (20 years old) and men's constraint curves were shifted towards lower marathon times (electronic supplementary material, table S2; figure 5*a*).

When we fitted a skew normal distribution to the recovery time data, accounting for which bird was singing and time relative to sunrise when estimating the constraint line improved model predictions over a simple model where bird ID and time relative to sunrise were not accounted for when estimating the constraint line. As above, the simple model was estimated to have lower out-of-sample accuracy (simple model: elpd_diff = −1846.9, se_diff = 89.2), as was the model run with a normal distribution (eldp_diff = −7867.8, se_diff = 407.1). Some birds' (e.g. bird 14) constraint lines were steeper and shifted towards lower values (i.e. higher performance), while other birds’ (e.g. bird 2) constraint lines were shallower (electronic supplementary material, table S3; figure 5*b*).

We compared two birds with large sample sizes and different constraint curves (figure 6). Bird 2's constraint line is less extreme than bird 18's, indicating that bird 2 is more constrained. A density plot of observed recovery times shows the difference in shape of the raw observations (figure 6*a*), and a point plot shows the estimated challenge (*y*-value) the birds experienced when singing individual songs (figure 6*b*).

## 9. Discussion: performance constraints in athletes and songbirds

We measured the skewness of 15 performance distributions and found that 14 of them skewed away from the direction of the constraint. Overall, these findings support the hypothesis that skewness is a signature of constraint. It is common knowledge that constraint can generate skew, and previous work has shown that athletic performance skews towards low performance [78]. Our conclusion that motor constraints cause performance distributions to skew away from the direction of constraint in a wide variety of athletic activities, however, appears to be novel.

Skewness was strong (|skewness| > 1) in 10 of the 15 distributions in our sample, and moderate (0.5 < |skewness| < 1) in four others, suggesting that constraint has a moderate to large influence on performance distributions in sports and bird song. The one exception, the women's 10 000 m race, was based on a small sample (*n* = 24) and had influential outliers (figure 2*h*). Like other distribution-level parameters, skewness tends to stabilize with sample size, and skewness analysis is more robust with larger samples (electronic supplementary materials), so larger samples are required before making conclusions about constraints in the women's 10 000 m race. The highest skewness values came from the bird song data. It may be that a history of selection for vocal performance has caused song to evolve to the point that it is highly constrained in Adelaide's warbler [15].

Our sports data come from trained athletes, raising the question of whether selection bias influenced our findings. We suspect constraints skew performance in most populations. Consider what would happen if a sample that was not selected for running performance (e.g. a large random sample of Canadian women aged 50–60) participated in a 1 km race in which effort was incentivized (e.g. with a monetary award based on finishing time). If each participant approached her own personal limit, the population must also approach its limit, which would generate skew. Empirical evidence in support of this intuition comes from the finding that the marathoners, who are a less elite population (i.e. a less biased sample) than the Olympians, still exhibited high skew. Further, the bird song data had the highest skewness, even though birds were sampled without regard to their singing abilities.

We generated posterior predictions from skew normal models to estimate constraints accounting for differences in resources (simulation), age/gender (marathon) classes, times (early morning versus later morning), and individuals (bird song; figures 4 and 5). The posterior predictions supported our *a priori* understanding of the effects of age and gender on marathon performance. The posterior predictions from the bird song analysis indicated that constraints vary among individuals and that singing Adelaide's warblers are less constrained after a warm-up period. We plotted behaviours on individuals' constraint curves and interpreted their location on the *y*-axis as the degree of challenge they experienced to produce the behaviour (figure 6*b*). Here, ‘challenge’ measures the degree to which constraint affects the behaviour, rather than the actor's subjective experience.

We interpret the constraint curves as estimates of the effect of motor constraints on performance. All else being equal, steeper curves indicate that the statistical population is more constrained. A single constraint curve may be influenced by constraints in various systems, including the muscular, nervous (both the peripheral nervous system and the central nervous system), and skeletal systems. These curves only describe the sample population, so changes in the population can change the constraint curves. For example, athletes' constraint curves may change with the adoption of new training techniques, diet or equipment. Similarly, non-human animals’ curves may change in response to environmental or evolutionary changes.

## 10. General discussion

The goals of this study were to evaluate and expand the toolkit for the analysis of performance constraints. A review of existing methods showed that none can estimate constraints with data from one behavioural trait in one population. A conceptual model of the evolution of performance in a constrained trait generates the prediction that the distribution of performance under constraint will be skewed, such that the long tail of the performance distribution points away from the constraint. Data from human athletes and singing birds supported this prediction. Our first set of simulations demonstrated that statistical models based on the skew normal distribution can be used to estimate constraint curves that characterize the relationship between performance (*x*) and challenge (*y*), with data from a single behavioural trait, while controlling for confounds. A second set of simulations examined how power and bias covary with effect size and sample size. We conclude that skewness and models based on the skew normal distribution are valuable tools for the identification and characterization of performance constraints. Although we have focused on sports and animal displays, this approach could also be applied to constrained behaviour in other domains.

The framework described in this paper contributes to a theory of performance under constraint. The conceptual model incorporates Podos's idea that motor constraint tends to increase with increasing performance, such that constraints are usually more like ramps than walls, and the lines that are traditionally used to represent constraints are more like isoclines than absolute limits [25]. The framework we propose also clarifies the meaning of the term ‘challenge,’ and permits its quantification. If we define challenge as the degree to which a constraint affects performance, the challenge of performing a particular behaviour can be quantified as that behaviours' location on the *y*-dimension of the constraint curve (figure 6*b*). This framing unifies the two definitions of performance described in the introduction. Performance is ‘an adaptive approach to a behavioural limit’ (the *x*-axes on figures 4, 5, 6*b*), which is highly correlated with ‘the degree of challenge experienced when performing the behaviour’ (the *y*-axis on figures 4, 5, 6*b*), because challenge increases as the population approaches the behavioural limit (figure 1).

When applied to individuals, as in the bird song dataset, the conceptual model describes how a sample of behaviours from an individual tends to skew when it interacts with the individual's own performance limit. It is possible to estimate and compare individuals' constraint curves with models based on the skew normal distribution (figures 5*b* and 6*b*). The *y*-coordinate of a single behaviour on the individual's constraint curve represents the degree of challenge required to generate that behaviour, relative to the individual's own constraint (figure 6*b*). The distance between the *x*-coordinate of a single behaviour, relative to the most extreme *x*-value (e.g. for the population) is an inverse estimate of performance. As with all behaviour-based performance metrics, it should be interpreted as an indirect measure of physiological performance [17]. When applied to populations of individuals, as in the sports datasets, the conceptual model describes how a sample of behaviours from a population will tend to skew when it interacts with a population-wide performance limit. The population-level approach produces only one constraint curve (that of the whole population). An individual's location on the constraint curve represents their performance. Mixed modelling, as in our bird song analysis, requires multiple samples of multiple individuals and permits estimation of both the individual-level and population-level constraint curves. As with other kinds of mixed models, it is possible to nest levels hierarchically. For example, signals occur within bouts, bouts occur within days, and days are sampled from individuals. This approach would permit analysis of constraint at each level.

The analytic approach we propose has the potential to help answer important questions in animal communication. One such question is whether to focus research attention on composite behaviours, like deviation scores, or the simple behaviours that comprise them [17]. If we want to know which kind of variation is more salient to receivers, only studies of receiver responses can provide satisfactory answers [43]. If, however, we wish to know which behaviour is more constrained, we could construct skew normal models and compare the variables' constraint curves. A similar approach could be used to address the concern that all edges of a performance distribution are equally likely to represent performance limits [21].

### 10.1. Open questions and limitations

There remain several unanswered questions about the proposed framework. For example, is it feasible to apply this framework to behavioural measures that cannot be well described with a skew normal distribution, such as those with discrete outcomes, or to multivariate cases in which multiple performance measures trade off? We analysed the multivariate bird song data in two steps—we first conducted a quantile regression to generate deviation scores, then analysed the distribution of deviation scores with a skew normal model. One shortcoming of this approach is that it fails to carry over uncertainty about the slope and intercept of the quantile regression into the skew-normal model. We are currently developing an alternative approach that models constraint as a two-dimensional surface.

A second question is how to model behavioural data that has been summarized, such as mean fast ball speed or free throw per cent. Taking the mean of samples repeatedly drawn from a distribution tends to produce a distribution of sample means that is normally distributed [79]. In the case of behavioural performance, even though this can be seen as sampling from separate individual distributions, there remains the possibility that summarizing the performance measures might alter the skewness of the distribution. This issue requires further investigation, but it is not a concern where individual performances are not summarized, as in the present data.

Skew is typical of distributions that are bounded on one side. We are interested in distributions that are bounded by a motor constraint, but other kinds of boundaries can also generate skew. A common example concerns proportional data, which is bounded by zero and one. Further research is necessary to disentangle the possible influences of such mathematical boundaries and performance constraints.

Uncontrolled variation can generate skewness in the absence of constraint. Consider an animal that performs a behaviour at two different levels (low and high), neither of which approaches a constraint. If the animal performs high more than low, for example, the distribution of performance would skew left even though there is no constraint. Similarly, contextual heterogeneity can exaggerate skew by altering a constraint, as seen in our bird song dataset. Male Adelaide's warblers sing with low vocal performance first thing in the morning. Their performance improves over the course of their dawn chorus as they ‘warm up’ (figure 5*c*; [51,55]). A model that does not include *time* as a covariate could over-estimate the skewness of their vocal performance. In addition to generating skew, uncontrolled variation can also mask skew (figures 2 and 3; [23,51]). Both skew-generating variation and skew-masking variation can be controlled by careful sampling and by including appropriate covariates in statistical models.

## 11. Conclusion

We found evidence to support the hypothesis that motor performance tends to skew away from the direction of constraint. We proposed a conceptual model of performance under constraint that can explain this pattern, and an approach to statistical modelling that uses the skew normal distribution to estimate constraints and degree of challenge based on a single kind of behavioural data. These models can facilitate the characterization and comparison of motor constraints on behaviour, including animal displays. Looking beyond the world of evolutionary biology, this approach may also have applications in other domains, including physiology and sports.

## Data Availability

Data and code are at github.com/tbonne/skewnormal. Supplementary material is available online [80].
